# Motor activity of centromere-associated protein-E contributes to its localization at the center of the midbody to regulate cytokinetic abscission

**DOI:** 10.18632/oncotarget.13206

**Published:** 2016-11-08

**Authors:** Akihiro Ohashi, Momoko Ohori, Kenichi Iwai

**Affiliations:** ^1^ Oncology Drug Discovery Unit, Pharmaceutical Research Division, Takeda Pharmaceutical Company Limited, Japan

**Keywords:** CENP-E, cytokinetic abscission, midbody localization, PRC1, CENP-E inhibitor

## Abstract

Accurate control of cytokinesis is critical for genomic stability to complete high-fidelity transmission of genetic material to the next generation. A number of proteins accumulate in the intercellular bridge (midbody) during cytokinesis, and the dynamics of these proteins are temporally and spatially orchestrated to complete the process. In this study, we demonstrated that localization of centromere-associated protein-E (CENP-E) at the midbody is involved in cytokinetic abscission. The motor activity of CENP-E and the C-terminal midbody localization domain, which includes amino acids 2659–2666 (RYFDNSSL), are involved in the anchoring of CENP-E to the center of the midbody. Furthermore, CENP-E motor activity contributes to the accumulation of protein regulator of cytokinesis 1 (PRC1) in the midbody during cytokinesis. Midbody localization of PRC1 is critical to the antiparallel microtubule structure and recruitment of other midbody-associated proteins. Therefore, CENP-E motor activity appears to play important roles in the organization of these proteins to complete cytokinetic abscission. Our findings will be helpful for understanding how each step of cytokinesis is regulated to complete cytokinetic abscission.

## INTRODUCTION

Cytokinesis, the final stage of cell division, distributes the replicated genome and other cellular components of the parent cell to the two daughter cells. Each daughter cell contains a single nucleus, a single centrosome, and a roughly equal share of cytoplasmic macromolecules and organelles [1–4]. Tetraploid cells, which arise by cytokinetic failure in certain cases, are proposed to promote aneuploidy, thereby contributing to the development of cancer [5]. Thus, accurate control of cytokinesis is critical for genomic stability across cell generations. Following chromosome segregation at anaphase, a cytokinetic cleavage furrow divides the two nascent daughter cells. These post-mitotic daughter cells, however, remain connected by an intercellular bridge containing antiparallel arrays of microtubules that overlap at a central region termed the midbody until cytokinetic abscission finally splits these cells apart. In the central spindle and midbody matrix, a number of proteins accumulate, including mitotic regulators, microtubule-bundling proteins, and lipid-raft or vehicle-trafficking proteins [6]. The temporally- and spatially-orchestrated dynamics of these components are critical to the completion of cytokinesis. Protein regulator of cytokinesis 1 (PRC1), a microtubule-bundling protein, directly binds microtubule proteins to form cross-bridges between antiparallel microtubules at the central spindle, which is required for further central spindle assembly and stability [7, 8]. Depletion of PRC1 disrupts the localization of critical cytokinesis effectors at the midbody, including the centralspindlin complex (kinesin motor protein MKLP1 and Rho-family GAP MgcRacGAP), the chromosomal passenger complex (aurora-B, borelin, INCENP, and survivin), and other midzone-associated proteins such as kinesin family member 4 (KIF4) [9, 10]. Thus, PRC1 not only promotes the formation of stable microtubules but also acts as a scaffold for structural components and signaling factors [4]. The kinesin motor protein KIF4, which localizes at the midbody in a PRC1-dependent manner, plays an important role in determining the size of antiparallel microtubule overlap at the anaphase central spindle. Specifically, KIF4, which is regulated by aurora-B phosphorylation, suppresses over-growth of microtubules to establish a narrow zone of interdigitating antiparallel microtubules, and also recruits a subset of protein phosphatase 2A to the spindle midzone [11–14]. Thus, signaling networks localized to the midbody via motor proteins appear critical for the organization and stabilization of a narrow zone of plus end overlap at the center of the midzone.

Centromere-associated protein-E (CENP-E) is a mitotic spindle motor protein of the kinesin superfamily [15]. During mitosis, CENP-E is localized to the kinetochore of chromosomes [15, 16] and controls chromosome alignment during metaphase by capturing microtubule plus ends at the kinetochore [17–20]. More recently, CENP-E was reported to transport the pole-proximal chromosomes toward the metaphase plate by a process dependent on tubulin post-translational modification [21]. After releasing the kinetochore during the transition to anaphase, CENP-E translocates to the central spindle and then to the midbody during the transition to cytokinesis [9, 22]. PRC1 interacts with CENP-E during late mitosis, and knockdown of PRC1 by small interfering RNAs (siRNAs) inhibits the translocation of CENP-E to the midbody [9]. These findings demonstrate that PRC1 is also involved in the accumulation of CENP-E at the midbody during late mitosis and cytokinesis by acting as a scaffold. This PRC1-mediated recruitment and accumulation of CENP-E at the midbody could play an important role in the progression of cytokinesis. However, the functions of CENP-E during cytokinesis remain to be elucidated.

In the current study, we demonstrate that localization of CENP-E to the midbody is involved in cytokinetic abscission. The motor activity of CENP-E and the C-terminal midbody localization domain, which includes RYFDNSSL at amino acids 2659–2666, are involved in the anchoring of CENP-E to the center of the midbody. Furthermore, CENP-E motor activity contributes to the intensive localization of PRC1 at the midbody during cytokinesis. Our studies reveal that regulation of the spatial organization of midbody-associated proteins such as PRC1 by CENP-E motor activity is required for efficient completion of cytokinesis. These findings will be helpful in understanding how each step of cytokinesis is regulated to complete cytokinetic abscission.

## RESULTS

### CENP-E contributes to cytokinetic abscission

**Figure 1:**
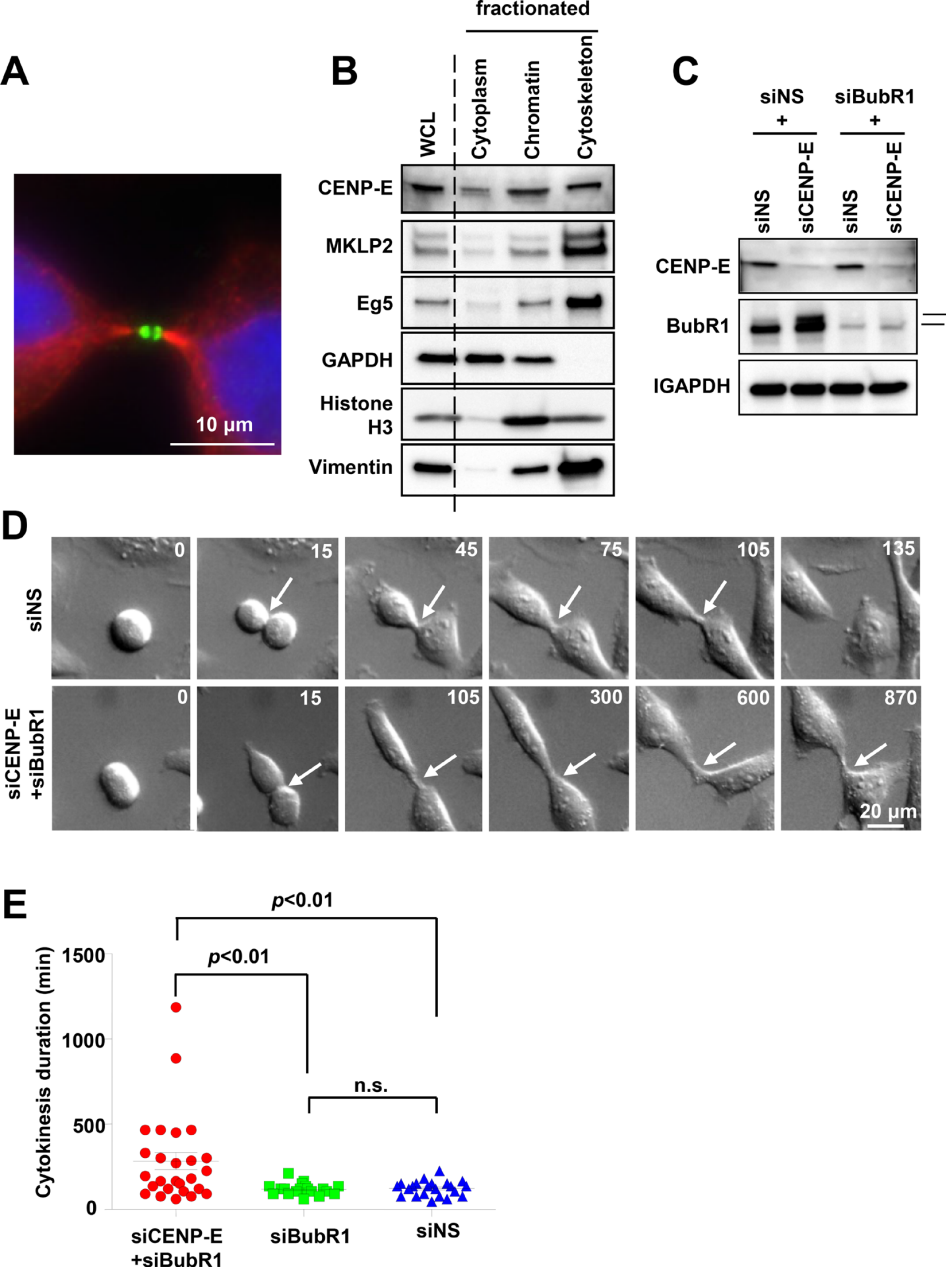
Effects of CENP-E on cytokinetic abscission in HeLa cells **A.** Localization of CENP-E at the midbody of telophase HeLa cells revealed by immunofluorescence staining with anti-CENP-E (green) and anti-α-tubulin (red). DNA was labeled with DAPI (blue). White bar indicates 10 μm. **B.** CENP-E expression in cytoplasmic, chromatin, and cytoskeletal fractions of HeLa cells. HeLa cells were collected 12 h after release from double-thymidine block and fractionated. Immunoblotting was performed for CENP-E and other mitotic kinesins (Eg5and MKLP2). GAPDH, histone H3, and vimentin were used as cytoplasm, chromatin, and cytoskeleton markers, respectively. **C.** Immunoblotting of CENP-E and BubR1 in HeLa cells transfected with siCENP-E and siBubR1. HeLa cells were treated with the indicated siRNAs for 24 h. The upper band in the BubR1 rectangle represents phosphorylated (activated) BubR1. GAPDH was used as the gel loading control. **D.** Representative time-lapse images of HeLa cells transfected with siCENP-E and siBubR1 (lower) or control siNS (upper). Frames were captured at the indicated time points (min) from onset to end of cytokinesis. White arrows indicate the cleavage furrow. White bar indicates 20 μm. **E.** Effects of CENP-E+BubR1 or BubR1 knockdown alone on cytokinesis duration estimated from time-lapse images (red, siCENP-E+siBubR1 HeLa cells (N = 26); green, siBubR1 HeLa cells (N = 19); blue, siNS HeLa cells (N = 22). Statistical analysis was performed using Student's t-test, and differences were considered significant at *p* < 0.01.

CENP-E functions in chromosome congression during metaphase by connecting microtubule plus ends to the chromosome kinetochores [15–18]. After release from the kinetochore during the transition from metaphase to anaphase [9, 22], CENP-E becomes concentrated at the central region of the midbody during cytokinesis [22, 23]. Indeed, CENP-E was detected at the midzone (Figure 1A) and in the cytoskeletal fraction of HeLa cells (Figure 1B). This cell cycle-dependent translocation suggests that CENP-E is involved not only in chromosome alignment but also in cytokinesis. To determine the function of CENP-E during cytokinesis, we performed time-lapse microscopy of HeLa cells following CENP-E knockdown using a targeted siRNA (Figure 1C and 1D).

Single knockdown of CENP-E (siCENP-E) activates the spindle assembly checkpoint during prolonged mitotic arrest to induce cell death [17, 24–29] (Figure 1C, upper band of BubR1 in the 2^nd^ lane). Thus, to evaluate the post-mitotic effects of CENP-E during cytokinesis, we knocked down the checkpoint regulator serine/threonine kinase BubR1 as well as CENP-E (siCENP-E + siBubR1 cells), a strategy that attenuates the spindle assembly checkpoint and allows cells to escape mitotic arrest. Time-lapse microscopy revealed that the duration of cytokinesis was significantly prolonged in siCENP-E + siBubR1 cells compared to siBubR1 and non-silencing control siRNA (siNS) cells (mean ± standard deviation, 283 ± 260 min versus 118 ± 34 and 122 ± 43 min, respectively; Figure 1E). Similar to control siBubR1 and siNS cells, however, cleavage furrow ingression was still observed in siCENP-E + siBubR1 cells. In addition, cytokinesis failure and subsequent polyploidy were rarely observed as only 1 out of 26 cells that underwent cytokinesis failed to complete it. Thus, CENP-E inhibition delays abscission but does not completely inhibit either this final process of cytokinesis or midbody formation to result in tetraploidy.

### Identification of the midbody localization domain of CENP-E

**Figure 2:**
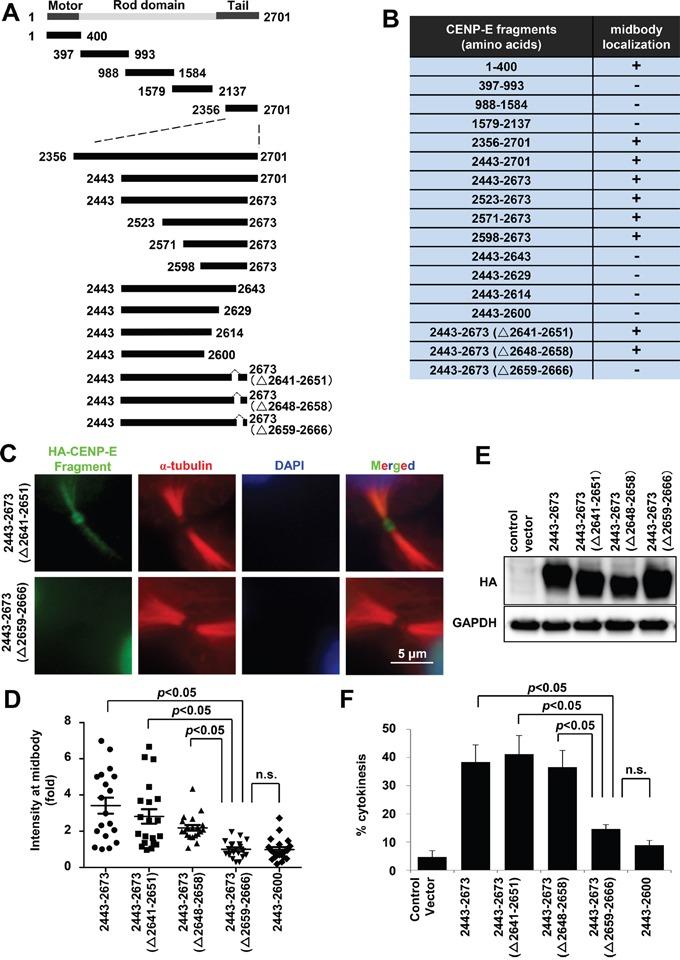
Mapping and characterization of the midbody localization domains of CENP-E **A.** Schematic diagrams of CENP-E fragments. Each fragment contains an HA-tag in the N-terminus. **B.** Summary of midbody localization by each CENP-E fragment. The ability (+) or inability (−) to localize to the midbody is shown. **C.** Representative IF images of different HA-tagged CENP-E fragments localized at the midbody. Green, red, and blue signals indicate HA-tagged CENP-E fragments, α-tubulin, and DAPI, respectively. **D.** Quantified signal intensity of CENP-E fragments at the midbody [IF intensity in a circle of 2-μm radius normalized to the mean intensity of cells transfected with the 2443–2673 (Δ2659–2666) fragment]. Statistical analysis was performed using Student's t-test. Differences were considered significant at *p* < 0.05. The n.s. indicates “not significant.” **E.** Immunoblotting of HA in HeLa cells transfected with the indicated HA-tagged CENP-E fragments. The indicated HA-tagged CENP-E fragments were transfected into HeLa cells. Twenty-four hours after transfection, the cells were collected for immunoblotting analysis. GAPDH was used as the gel loading control. All fragments were robustly expressed. **F.** Fraction (%) of cytokinetic HeLa cells expressing the indicated CENP-E fragments (mean ± standard deviation). Statistical analysis was performed using Student's t-test. Differences were considered significant at *p* < 0.05. The n.s. indicates “not significant.”

To identify the midbody localization domain(s) of CENP-E, localization of five HA-tagged CENP-E fragments at the midbody was evaluated in HeLa cells by immunofluorescence (IF) using an anti-HA (Figure 2A and 2B). Both the N-terminal motor region (amino acids 1–400) and the C-terminal tail region (amino acids 2356–2701) were localized to the midbody in HeLa cells. We performed further IF-based mapping of the C-terminal CENP-E (Figure 2A and 2B), which demonstrated that C-terminal CENP-E fragments without RYFDNSSL at amino acids 2659–2666 exhibited much fainter IF at the midbody (Figure 2B, 2C, and Supplementary Figure S1). The midbody localization of each fragment was quantified by the IF intensity in a circle of 2-μm radius centered at the midbody (Figure 2D and Supplementary Figure S2) and normalized to the mean intensity of the 2443–2673 (Δ2659–2666) fragment. Quantification demonstrated that the signal intensities of all the fragments without RYFDNSSL were significantly reduced at the midbody (Figure 2D), while robust expression of each (HA-tagged) fragment was confirmed by immunoblotting (Figure 2E). These findings suggest that RYFDNSSL at 2659–2666 of the C-terminal facilitates efficient localization of CENP-E at the midbody. The expression of exogenous C-terminal CENP-E fragments containing the RYFDNSSL sequence at amino acids 2659–2666 also significantly increased the fraction of cytokinetic cells compared to cells transfected with control vector, from 5% (control) to 38.3% by amino acids 2443–2673, to 41.2% by 2443–2673 (Δ2640–2652), and to 36.6% by 2443–2673 (Δ2648–2659) (Figure 2F). On the contrary, exogenous expression of C-terminal CENP-E fragments without RYFDNSSL did not induce a substantial elevation in the number of cytokinetic cells, to only 8.9% by amino acids 2443–2600 and to 14.7% by 2443–2673 (Δ2641–2651) (Figure 2F). Although all transfected cells exhibited intercellular bridge formation with an ingressed cleavage furrow like control cells (Figure 2C, Supplementary Figures S1, S2), expression of exogenous CENP-E C-terminal fragments with the RYFDNSSL sequence delayed cytokinetic abscission and splitting of the daughter cells in a dominant-negative manner.

**Figure 3:**
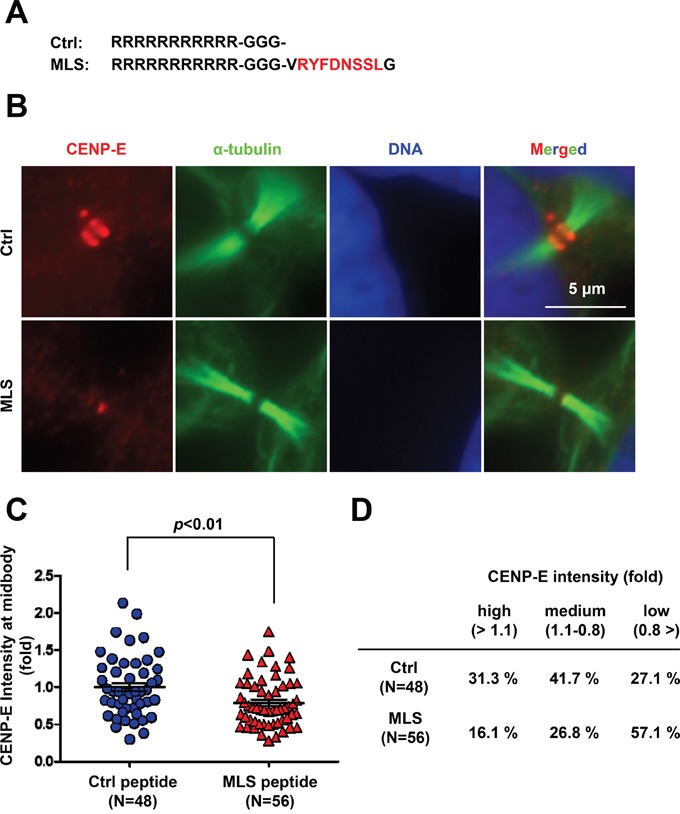
Treatment with the C-terminal midbody localization sequence (MLS) peptide reduces endogenous CENP-E localization at the midbody **A.** The MLS sequence was conjugated to cell-permeable Arg^11^. **B.** Immunofluorescence of CENP-E in HeLa cells treated with the MSL peptide or control peptide (100 μM) for 24 h. Immunofluorescence staining was performed with anti-CENP-E (red) and anti-α-tubulin (green). DNA was labeled with DAPI (blue). White bar indicates 5 μm. **C** and **D.** Quantification of CENP-E immunofluorescence in HeLa cells treated with the MLS peptide or control peptide. The midbody localization of CENP-E was quantified by the IF intensity in a circle of 1.5-μm radius at the midbody (control peptide; N = 48, MLS peptide; N = 56) normalized to the mean intensity of control peptide-treated cells. Statistical analysis was performed using Student's t-test. Differences were considered significant at *p* < 0.05. The quantified CENP-E signals were categorized into three groups based on the relative IF intensity: high (>1.1), medium (1.1–0.8), and low (<0.8).

To examine whether the 8-amino acid peptide RYFDNSSL modulates localization of endogenous CENP-E protein at the midbody, we synthesized a 10-amino acid peptide (amino acids 2658–2667) with RYFDNSSL at the center and conjugated this peptide to a cell membrane-permeable polyarginine (Arg^11^) sequence at the N-terminus [30] (Figure 3A). HeLa cells were treated with 100 μM of this fusion peptide containing the 8-amino acid RYFDNSSL sequence (termed the midbody localization sequence, MLS) or a control peptide for 24 h, followed by immunofluorescence staining of CENP-E (Figure 3B). The midbody localization of endogenous CENP-E was quantified by the IF intensity in a circle of 1.5-μm radius centered at the midbody (Figure 3C and Supplementary Figure S3) normalized to the mean intensity of control peptide-treated cells. Treatment with the MLS-containing peptide resulted in significantly diminished IF intensity from endogenous CENP-E at the midbody compared to the control peptide (Figure 3A-3C). Cells subjected to CENP-E quantification at the midbody by IF staining were categorized into three groups based on relative IF intensity; high (>1.1), medium (1.1–0.8), and low (<0.8) (Figure 3D). Of MSL peptide-treated cells, 16.1% were high, 26.8% medium, and 57.1% low intensity, whereas among control peptide-treated cells, 31.3% were high, 41.7% medium, and only 27.1% low intensity (Figure 3D), demonstrating that the MLS peptide decreases endogenous CENP-E localization at the midbody through a dominant-negative effect.

Mapping studies revealed that CENP-E possesses two domains for midbody localization (Figure 2B), one in the N-terminal motor region (1–400) and the other in the C-terminal tail region (2659–2666). The MLS peptide is expected to interfere with the C-terminal midbody localization domain but not with the N-terminal domain. Thus, the endogenous CENP-E protein still localized to the midbody after MLS peptide treatment may depend on the N-terminal motor region. Unlike exogenous expression of the CENP-E fragments (Figure 2C-2F), however, accumulation of cytokinetic cells was not observed with MLS peptide treatment (data not shown), suggesting that the biological activity of this peptide is not high enough to delay cytokinesis abscission.

### CENP-E motor activity is involved in anchoring CENP-E at the midbody dark zone

**Figure 4:**
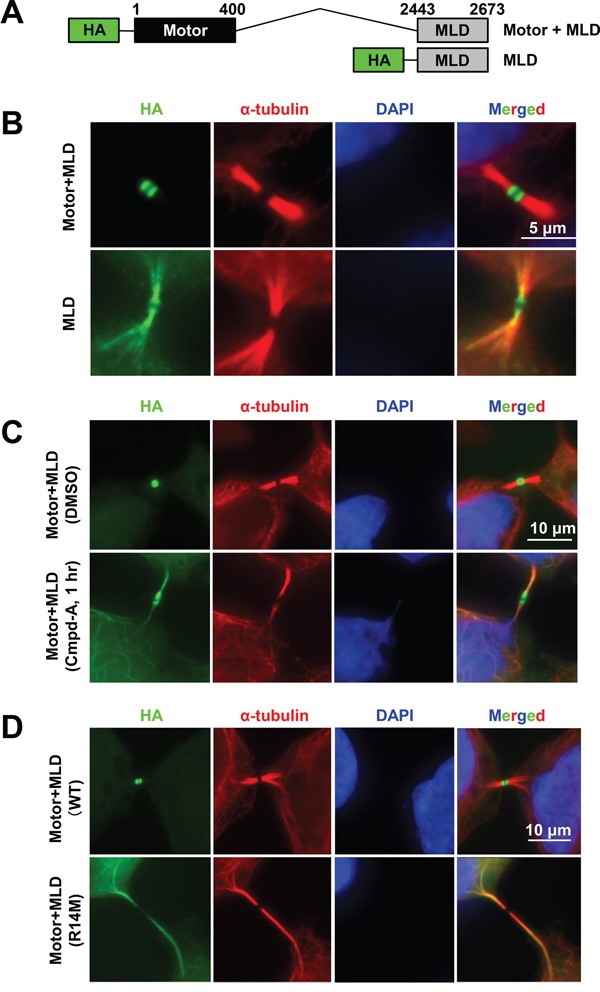
CENP-E motor activity is required for intense midbody accumulation **A.** Schematic of CENP-E fragments consisting of the motor region and MDL (Motor+MLD; upper) or just the MLD (lower). **B.** Effect of the CENP-E motor domain on midbody localization. Representative IF images of the Motor+MLD and MLD peptides at the midbody. Immunofluorescence was performed with anti-HA (green) and anti α-tubulin (red). DNA was labeled with DAPI (blue). Scale bar, 5 μm. **C** and **D.** Effect of CENP-E ATPase activity on midbody localization. ATPase activity of the CENP-E motor domain was attenuated by Cmpd-A (a CENP-E inhibitor) (C) or replacement of amino acids in the ATP-binding pocket of the motor domain (D). Representative images showing CENP-E fragment localization to the midbody. Immunofluorescence was performed with anti-HA (green) and anti-α-tubulin (red). DNA was labeled with DAPI (blue). Scale bars, 10 μm.

The other CENP-E midbody localization domain is located in proximity to the N-terminal motor domain, which moves CENP-E forward along microtubules to their plus ends. To investigate the contribution of this CENP-E domain to midbody localization, we generated a conjugated CENP-E fragment of the C-terminal midbody localization domain (MLD; amino acids 2443–2673) with the N-terminal motor domain (Motor; amino acids 1–400; Figure 4A). Since full-length CENP-E is a large molecular weight protein (2701 amino acids), functional analysis using full-length CENP-E plasmids is technically difficult due to low transfection efficiency. Thus, plasmids encoding CENP-E fragments were used for the following transfection experiments. Immunofluorescence of HeLa expressing these exogenous fragments revealed that although the MLD alone localized to a broad zone of the midbody, the motor-conjugated MLD (Motor+MLD) localized intensely at the “dark zone” [31], a narrow region at the center of the midbody in between the dense microtubule bundles (Figure 4B). For further evaluation of the motor activity at the dark zone, we used a small molecule compound that inhibits the ATPase activity of the N-terminal motor domain of CENP-E, Compound-A (Cmpd-A), that we developed previously [32, 33]. Motor+MLD-expressing HeLa cells were treated for 1 h with 200 nM Cmpd-A or dimethyl sulfoxide (DMSO) as a control, followed by IF staining with anti-HA antibody. Treatment with Cmpd-A redistributed Motor+MLD to a broader region along the microtubule filaments, whereas the highly intense midbody localization was maintained in control cells treated with DMSO (Figure 4C). We also confirmed more diffuse localization of an ATPase-attenuated Motor+MLD in which the arginine at position 14 of the CENP-E ATPase active site was replaced by methionine (R14M) [34] (Figure 4D).

**Figure 5:**
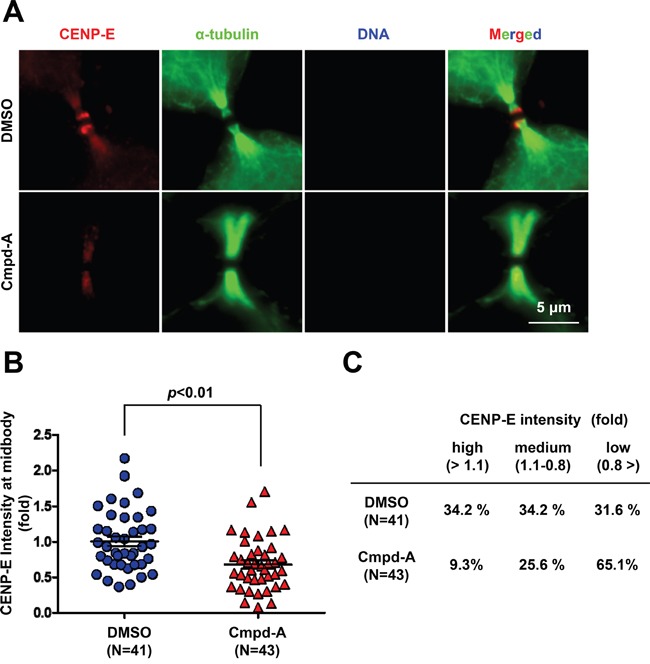
Treatment with the CENP-E inhibitor reduces endogenous CENP-E localization at the midbody **A.** Immunofluorescence of CENP-E in HeLa cells treated with Cmpd-A (200 nM) or DMSO for 2 h. Immunofluorescence staining was performed with anti-CENP-E (red) and anti-α-tubulin (green). DNA was labeled with DAPI (blue). White bar indicates 5 μm. **B** and **C.** Quantification of CENP-E immunofluorescence in HeLa cells treated with Cmpd-A or DMSO. The midbody localization of CENP-E was quantified by the IF intensity in a circle of 1.5-μm radius at the midbody (DMSO; N = 41, Cmpd-A; N = 43) normalized to the mean intensity of DMSO-treated cells. Statistical analysis was performed using Student's t-test. Differences were considered significant at *p* < 0.05. The quantified CENP-E signals were categorized into three groups based on the relative IF intensity: high (>1.1), medium (1.1–0.8), and low (<0.8).

We next examined whether inhibition of CENP-E motor activity affects midbody localization of endogenous CENP-E. HeLa cells were treated for 2 h with 200 nM Cmpd-A or DMSO as a control, followed by IF with an anti-CENP-E antibody (Figure 5A). The midbody localization of endogenous CENP-E was quantified by the IF intensity in a circle of 1.5-μm radius centered at the midbody (Figure 5B and Supplementary Figure S4) normalized to the mean intensity of DMSO-treated cells. Treatment with Cmpd-A significantly diminished the signal intensity from endogenous CENP-E protein at the midbody compared to treatment with DMSO (Figure 5A and 5B). As in Figure 3, cells immunostained for CENP-E with or without Cmpd-A treatment were also categorized into three groups based on relative IF intensity at the midbody: high (>1.1), medium (1.1–0.8), and low (<0.8) (Figure 5C). Only 9.3% of Cmpd-A-treated cells exhibited high intensity and 25.6% medium intensity, while 65.1% showed low intensity. In contrast, 34.2% of DMSO-treated cells exhibited high intensity, 34.2% medium, and only 31.6% low intensity (Figure 5C). These results demonstrate that CENP-E ATPase activity is required for anchoring CENP-E at the dark zone in the midbody.

### PRC1 interacts with the N- and C-terminal CENP-E domains and modulates CENP-E motor activity *in vitro*

**Figure 6:**
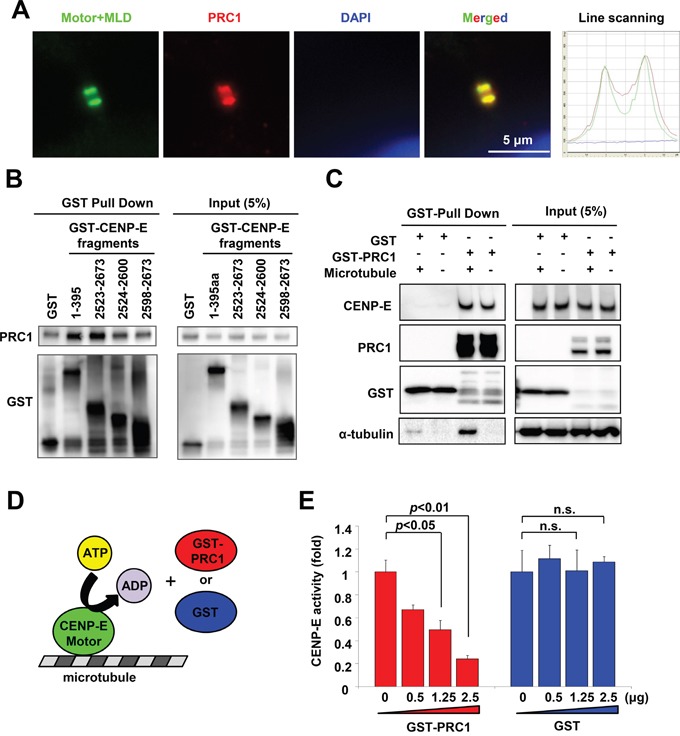
PRC1 interacts with CENP-E and modulates CENP-E ATPase activity *in vitro* **A.** The CENP-E Motor+MLD fragment colocalized with PRC1 at the midbody. Representative images of Motor+MLD colocalized with PRC1 at the midbody. Immunofluorescence was performed with anti-HA (green) and anti-PRC1 (red). DNA was labeled with DAPI (blue). Histogram of the line scanning indicates the intensity of each signal. **B.** N- and C-terminal CENP-E fragments interacted with recombinant PRC1 *in vitro*. Recombinant His-tagged PRC1 proteins and the indicated GST-tagged CENP-E fragment were used for GST pull-down assays (left, GST pull-down; right, 5% input). Immunoblotting with anti-PRC1 or anti-GST antibodies was performed. **C.** Interaction of PRC1 and endogenous CENP-E. The GST-tagged PRC1 recombinant proteins, microtubules, and cell lysates from 293T cells were used for GST pull-down assays (left, GST pull-down; right, 5% input). Immunoblotting with anti-CENP-E, anti-GST, or anti-α-tubulin antibodies was performed. **D.** Schematic diagram of *in vitro* enzymatic assay for CENP-E motor activity. **E.** PRC1 suppresses CENP-E motor activity *in vitro*. The X-axis indicates the concentration of GST-PRC1 (red) or GST (blue), and the Y-axis indicates the ATPase activity of the CENP-E motor domain. Statistical analysis was performed using Student's t-test. Differences were considered significant at p < 0.05.

The midbody accumulates many cytokinetic proteins, such as CENP-E, PRC1, MKLP1 RacGAP1, and Aurora-B (Supplementary Figure S5A) [6, 31], which are categorized into three groups according to the part of the midbody at which they localize: the bulge, the dark zone, or the flanking zone. The orchestrated localization and dynamics of these cytokinetic proteins at the midbody are critical for the highly coordinated rearrangement of cytoskeleton and membrane required for cytokinetic abscission [35]. We thus investigated whether CENP-E contributes to the organization of other cytokinetic proteins at the midbody during cytokinesis. Immunofluorescence revealed that Motor+MLD colocalized with the dark zone protein PRC1 in the midbody (Figure 6A) but not with the bulge proteins MKLP1 and RacGAP1 or the flanking protein aurora-B (Supplementary Figure S5B). A cell-free pull-down assay revealed that the recombinant full-length PRC1 protein bound to both the N-terminal (amino acids 1–395) and C-terminal (amino acids 2523–2673) CENP-E fragments (Figure 6B, Supplementary Figure S6A, S6B). A pull-down assay using 293T cell lysate also detected binding of endogenous CENP-E with recombinant PRC1 (Figure 6C). Added microtubules did not affect the binding affinity between CENP-E and PRC1, although exogenous microtubules increased microtubule-bound PRC1. Given that PRC1 binds to microtubules directly *in vitro* without CENP-E [36], microtubule-binding appears to be dispensable for the physical interaction between CENP-E and PRC1. Consistent with these findings, our mapping studies demonstrated that the PRC1-binding domain of CENP-E does not fully coincide with its midbody localization domain. The fragment with amino acids 2598–2673 accumulates at the midbody (Figure 2B and Supplementary Figure S1) but shows substantially attenuated PRC1 binding (Figure 6B). Taken together, CENP-E and PRC1 bind directly in a microtubule-independent manner, but both do associate independently with microtubules.

To clarify the effects of PRC1 on CENP-E function, we next performed a cell-free enzymatic assay to measure *in vitro* CENP-E motor activity in the presence and absence of recombinant PRC1 (GST-PRC). Recombinant GST protein was used as a control. CENP-E moves along microtubule filaments powered by ATP hydrolysis. Therefore, *in vitro* CENP-E motor activity was assessed by measuring the amount of inorganic phosphate generated by CENP-E ATPase activity (Figure 6D). As shown in Figure 6E, CENP-E ATPase activity was significantly suppressed by GST-PRC1 in a dose-dependent manner (red bars) but not by control GST (blue bars). PRC1 molecules exhibit a coil formation along the microtubules, and protrude perpendicular to the microtubule fiber direction [7]. It is possible that PRC1 coiled on the microtubules interacts with the motor domain of CENP-E *in vitro*, and that CENP-E/PRC1 complex formation reduces the forward velocity of the CENP-E motor on plus-ended microtubules.

### CENP-E motor activity is involved in anchoring PRC1 at the midbody dark zone

**Figure 7:**
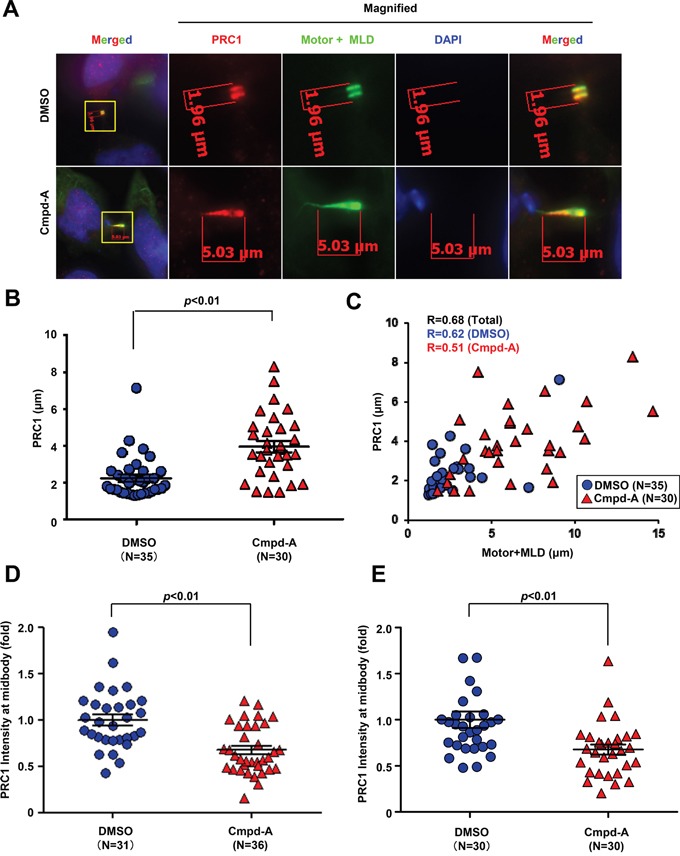
CENP-E modulates PRC1 localization to the midbody by its motor activity **A.** Effect of CENP-E motor activity on localization of PRC1 at the midbody. HeLa cells were transfected with CENP-E Motor+MLD. Twenty-four hours after transfection, cells were treated with 200 nM Cmpd-A (lower) or vehicle (DMSO, upper) for 1 h. Following fixation, cells were stained with anti-PRC1 (red) and anti-HA (green). DNA was labeled with DAPI (blue). **B.** Quantification of PRC1 dispersion (length) at the midbody. The graph shows the distance of PRC1 at the midbody based on immunofluorescence analysis. Statistical analysis was performed using Student's t-test. Differences were considered significant at *p* < 0.05. **C.** Correlation of Motor+MLD and PRC1 dispersion (distances) at the midbody. The graph shows the quantitative distance of PRC1 (Y-axis) and the Motor+MLD (X-axis) based on immunofluorescence analysis. **D.** Quantification of PRC1 immunofluorescence in HeLa cells treated with Cmpd-A or DMSO. The midbody localization of PRC1 was quantified by the IF intensity in a circle of 1.5-μm radius at the midbody (DMSO; N = 31, Cmpd-A; N = 36) normalized to the mean intensity of DMSO-treated cells. Statistical analysis was performed using Student's t-test. Differences were considered significant at *p* < 0.05. **E**. Quantification of PRC1 immunofluorescence in siBubR1-transfected HeLa cells treated with Cmpd-A or DMSO. Twenty-four hours after siBubR1 transfection, HeLa cells were treated with 200 nM Cmpd-A or DMSO for 24 hours. The midbody localization of PRC1 was quantified by the IF intensity in a circle of 1.5-μm radius at the midbody (DMSO; N = 30, Cmpd-A; N = 30) normalized to the mean intensity of DMSO-treated cells. Statistical analysis was performed using Student's t-test. Differences were considered significant at p < 0.05.

Next, we examined whether CENP-E motor activity contributes to the localization of PRC1 at the midbody. HeLa cells expressing the Motor+MLD fragment of CENP-E were treated with Cmpd-A or vehicle for 1 h, followed by immunofluorescence staining of PRC1. As shown in Figure 7A (upper panels), control vehicle-treated (DMSO) cells exhibited intense colocalization of Motor+MLD with PRC1 in the dark zone, while treatment with Cmpd-A broadly diffused PRC1 as well as Motor+MLD across the dark zone on midbody filaments (Figure 7A, lower panels). Quantitative evaluation confirmed that PRC1 localization at the midbody was significantly dispersed (elongated) in response to Cmpd-A treatment (*p* < 0.01, mean ± standard deviation, 2.2 ± 1.1 μm with DMSO and 3.9 ± 1.8 μm with Cmpd-A; Figure 7B). Furthermore, the extents of PRC1 and Motor+MLD dispersal along the microtubule bundles were significantly (*p* < 0.01) correlated based on Pearson product–moment correlation coefficient analysis [R = 0.68 in total (N = 67), 0.62 in DMSO (N = 36), and 0.51 in Cmpd-A (N = 31); Figure 7C, blue circles; control, red triangles; Cmpd-A]. As demonstrated in Figures 3C and 5B, the midbody localization of endogenous PRC1 was also quantified by the IF intensity in a circle of 1.5-μm radius centered at the midbody (Figure 7D and Supplementary Figure S7) normalized to the mean intensity of DMSO-treated cells. We also assessed the effects of Cmpd-A on the midbody localization of endogenous PRC1 in siBubR1 HeLa cells, in which the Cmpd-A-mediated SAC activation and the prolonged mitotic arrest were bypassed (Figure 7E). Treatment with Cmpd-A significantly diminished the signal intensity of endogenous PRC1 at the midbody compared to DMSO treatment in both normal HeLa and siBubR1 HeLa cells (Figure 7D and 7E). Thus, CENP-E motor activity is involved in the anchoring of PRC1 to the dark zone of the midbody to establish a scaffold for subsequent recruitment of cytokinetic signaling proteins.

**Figure 8:**
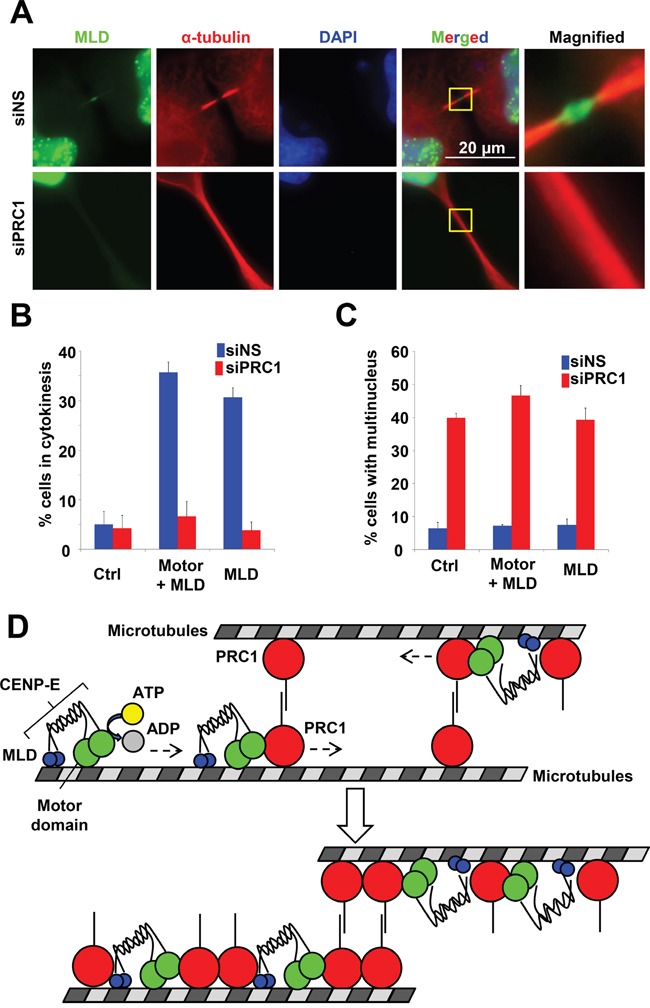
Effect of PRC1 on CENP-E localization to the midbody **A.** PRC1 knockdown attenuated MLD localization to the midbody. HeLa cells were transfected with the MLD plasmid of CENP-E 24 h after siNS (upper) or siPRC1 (lower) treatment. Twenty-four hours after transfection, cells were fixed for immunofluorescence staining with anti-HA (green) and anti-PRC1 (red). DNA was labeled with DAPI (blue). **B.** Fraction (%) of HeLa cells expressing the indicated CENP-E fragments during cytokinesis in the presence of siNS (blue) or siPRC1 (red). **C.** Fraction (%) of multinucleated HeLa cells expressing the indicated CENP-E fragments in the presence of siNS (blue) or siPRC1 (red). **D.** Schematic of CENP-E motor activity associated with the PRC1 localization at the midbody.

To examine the effect of PRC1 on MLD accumulation at the midbody, the MLD plasmid was transfected into HeLa cells with PRC1 siRNA (siPRC1) or non-silencing siRNA (siNS). As shown in Figure 8A, the MLD signal at the midbody was markedly fainter in siPRC1-transfected cells compared to siNS-transfected controls. The IF of endogenous CENP-E at the midbody was also fainter in siPRC1-transfected cells compared to controls (Supplementary Figure S8). The dark zone was not observed at the intercellular bridge in siPRC1-transfected cells (Figure 8A), presumably resulting in failed accumulation of MLD and full-length CENP-E at the midbody. Furthermore, while expression of exogenous MLD and Motor-MLD increased the number of cells in the cytokinetic phase (Figures 2F and 8B, blue bars), siPRC1 significantly reduced the number of cytokinetic cells in both MLD- and Motor+MLD transfection groups (Figure 8B, red bars). Alternatively, siPRC1 elevated multinucleation in MLD- and Motor+MLD-expressing cells as well as in control cells (Figure 8C), demonstrating that aberrant antiparallel microtubule formation caused by siPRC1 results in failure to bypass prolonged cytokinesis via the dominant-negative effects of exogenous MLD or Motor+MLD.

## DISCUSSION

In this study, we investigated the molecular function of CENP-E at the midbody during cytokinetic abscission. Our data revealed that the motor activity of CENP-E and the C-terminal midbody localization domain containing RYFDNSSL are involved in anchoring CENP-E to the center (dark zone) of the midbody. Furthermore, CENP-E motor activity is required for intensive localization of both PRC1 and CENP-E to the midbody during cytokinesis. Biochemical analysis also revealed that CENP-E physically associates with PRC1, and that PRC1 modulates the ATPase activity of the CENP-E motor domain *in vitro*. These findings suggest that if PRC1 binds to a microtubule ahead (more toward the + end) of CENP-E, CENP-E may slide PRC1 molecules along the microtubule using CENP-E motor activity (Figure 8D). These functional interactions between CENP-E and PRC1 at the midbody could play important roles in organizing midbody structures around antiparallel microtubules for completing cytokinetic abscission.

Although CENP-E has been reported to accumulate at the midbody in cytokinesis [37], the functional roles of CENP-E in midbody assembly are largely unknown. Immunofluorescence-based mapping indicated that the N-terminal motor region (amino acids 1–400) and C-terminal tail region (amino acids 2357–2701) of CENP-E are involved in its midbody localization during mitosis (Figure 2). It has been reported that the N-terminus (amino acids 1–540) and C-terminus (amino acids 2109–2663) of CENP-E bind directly to microtubules *in vitro* [37]. Microscopic analysis revealed that CENP-E fragments including MLD also localized along microtubule filaments at the intercellular bridge (Figure 3). Taken together, direct binding of CENP-E to central spindle microtubules appears to be involved in its midbody localization, and the 8-amino acid RYFDNSSL sequence of CENP-E at the C-terminus may contribute to its direct binding with microtubules.

Like CENP-E, proteins such as PRC1, motor proteins (KIF4, MKLP1, MKLP2, and KIF14), and mitotic kinases (PLK1 and aurora-B) accumulate at the midbody in cytokinesis. The microtubule-bundling protein PRC1 forms cross-bridges between antiparallel microtubules for central spindle assembly and stability [7]. Two kinesins, KIF4 and the centralspindlin complex (kinesin motor protein MKLP1 and Rho-family GAP MgcRacGAP), work in concert with PRC1 to organize and stabilize a narrow zone of microtubule plus end overlap at the center of the midzone in a process regulated by aurora-A and Plk1 [4]. It has been reported that KIF4 regulates the microtubule overlap size of the anaphase central spindle by suppressing microtubule over-growth, thereby establishing a narrow zone of interdigitating antiparallel microtubules. Furthermore, KIF4 recruits a subset of protein phosphatase 2A to the spindle midzone [11–14]. Recent studies have demonstrated that mitotic kinesin Eg5 drives PRC1 along microtubules *in vitro* [7]. Supporting previous studies [9], our data revealed that PRC1 binds to the N- and C-terminus of CENP-E, and that microtubule interaction appears dispensable for this interaction (Figure 6). We also demonstrated that CENP-E motor activity contributes to the high accumulation of PRC1 at the dark domain of the midbody. Furthermore, it has been reported that PRC1 binds to several motor proteins in late-phase mitotic cells, including KIF4, MKLP1, MKLP2, KIF14, and CENP-E [9, 38]. More recently, Lee *et al.* demonstrated that direct interaction of PRC1 with the centralspindlin complex, which includes MKLP1, contributes to a mechanism for recovery of perturbations caused by excess tension from cortical pulling forces [39]. Given that this interaction is critical for central spindle formation, the structural integrity of the protein complex at the midbody appears vital not only for cytokinesis but also in the response to microtubule dynamics. On the contrary, the N-terminal PRC1 region, a PRC1 dimerization domain, is shared by KIF4, MKLP1, and CENP-E for binding, but PRC1 binds separately to these three motor proteins [9]. These findings suggest that although various mitotic kinesins regulate midbody accumulation of PRC1, different kinesins play unique roles in the formation of cross-bridges between antiparallel microtubules at the midbody.

In the current study, we demonstrate that CENP-E is involved in the accumulation of PRC1 at the dark region of the midbody. Loss of CENP-E function in cytokinesis results in a phenotype distinct from loss of PRC1 or its regulator KIF4. Suppression of KIF4 expression inhibits PRC1 translocation and midzone formation, resulting in the failure of cytokinesis and polyploidy [40]. On the contrary, siRNA-mediated CENP-E knockdown resulted in prolonged cytokinesis (Figure 1), and overexpression of CENP-E fragments also caused accumulation of cytokinetic cells in a dominant-negative manner. Furthermore, disorganized central spindle formation and polyploidy by failed cytokinesis were rarely observed in these cells. Although the effects of CENP-E depletion are not as severe as those of KIF4 depletion, these observations suggest that CENP-E is also an important regulator of final cytokinetic abscission. We suggest that CENP-E may contribute to the accumulation of key components at the dark zone, such as the ESCRT complex [41, 42], by first recruiting PRC1, which acts as a multiprotein binding platform.

We also demonstrate the physiological contribution of CENP-E to cytokinesis; CENP-E inhibition by siRNA knockdown significantly delayed cytokinetic abscission (Figure 1). On the contrary, it has been reported that a chromosome bridge generated by chromosome missegregation delays cytokinetic abscission via aurora-B-mediated abscission checkpoint machineries [43]. Given that CENP-E is also involved in chromosome alignment [17–20], it possible that the delayed abscission observed in this study could derive from the indirect effects of chromosome misalignment rather than a direct involvement of CENP-E in cytokinesis. Although this possibility cannot be completely excluded, the following findings support the direct involvement of CENP-E in cytokinetic abscission. First, CENP-E localized at the dark zone of the midbody during cytokinesis (Figure 1). Second, elevation of cytokinetic cells and midbody localization of CENP-E fragments were correlated (Figure 2). Third, CENP-E bound directly to the cytokinesis-regulatory protein PRC1 (Figure 6). Fourth, CENP-E and PRC1 appeared to interact functionally in cytokinetic regulation (Figures 6–8). Further studies will be needed to determine the contributions of CENP-E to cytokinetic abscission at the molecular level.

In conclusion, we demonstrate that CENP-E motor activity is involved in cytokinetic abscission by anchoring CENP-E and its binding partner PRC1 at the center of the midbody. CENP-E appears to facilitate final abscission by mediating the assembly of a number of midbody factors. These spatial and temporal controls of midbody factors by CENP-E could be critical for cytokinetic abscission.

## MATERIALS AND METHODS

### Cell cultures

HeLa cells were purchased from American Type Culture Collection (Manassas, VA, USA) and cultured in Dulbecco's Modified Eagle's Medium supplemented with 10% fetal bovine serum.

### Plasmid construction

HA-tagged CENP-E fragments, GST-tagged CENP-E fragments, GST-tagged PRC1, and His-tagged PRC1 were cloned by the GATEWAY system according to the manufacture's protocols (Invitrogen). Oligonucleotide primers for the cDNA fragments were designed with flanking attB1 or attB2 sites for insertion into the GATEWAY donor vector pDONR201/Zeo (Invitrogen). Subsequently, these fragments were transferred into *Escherichia coli* expression vectors pDEST15 and pDEST17 or into the mammalian expression vector phCMV2 (Genlantis, San Diego, CA, USA), each of which was modified to be compatible with the GATEWAY system. The primer sequences used in this study are provided in Supplementary Table S1. All genetic recombination studies were performed in accordance with protocols approved by the Technical Committee for Genetic Recombinant Experiments at Takeda Pharmaceutical Co., Ltd. (Experimental Protocol No. 2217; Osaka, Japan).

### Immunofluorescence

Immunofluorescence was performed as described previously [44]. The following primary antibodies were used: anti-CENP-E (1:100 dilution; sc22790; Santa Cruz Biotechnology), anti-α-tubulin (1:1000 dilution; T9026; Sigma-Aldrich), anti-PRC1 (1:100 dilution; ab21437; Abcam), and anti-HA fluorescein (1:50 dilution; 3F10; Roche). Immunolabeling was visualized using the following secondary antibodies: Alexa Fluor 488-conjugated goat anti-mouse IgG (1:500 dilution; A11011; Invitrogen), Alexa Fluor 594-conjugated goat anti-mouse IgG (1:500 dilution; A11032; Invitrogen), and Alexa Fluor 594-conjugated goat anti-rabbit IgG (1:500 dilution; A11012; Invitrogen). Images were captured with a Plan-Apochromat 100× oil-immersion lens on an Axiovert 200M microscope (Carl Zeiss, Jena, Germany).

### Time-lapse imaging and analysis

HeLa cells were cultured in 24-well culture plates with glass bottoms (MatTek Corp., Ashland, MA, USA) and transfected with siRNA oligos targeting the indicated genes: BubR1 (siGENOME SMARTpool, Cat# M-004101-02, Thermo Scientific Darmacon), CENP-E (siGENOME SMARTpool, Cat# M—003252-02, Thermo Scientific Darmacon), and PRC1 (siGENOME SMARTpool, Cat# M-019491-00, Thermo Scientific Darmacon). A non-silencing (NS) siRNA was used as a control (siTRIO Negative Control, Cat# S6C-0120, B-Bridge International Inc.). The siRNA-transfected HeLa cells were cultured in Dulbecco's Modified Eagle's Medium supplemented with 10% fetal bovine serum, and incubated for at least 3 h before image capture on a microscope stage-mounted humidified chamber (Incubator XL S1, Carl Zeiss) at 37°C in 5% CO_2_. Phase-contrast images were captured every 15 min for 24 h (12–36 h after transfection). Cells were imaged using an Axiovert 200M microscope (Carl Zeiss) equipped with an EC Plan NeoFluar 20× lens (Carl Zeiss). Images were acquired and processed using AxioVision 4.5 software (Carl Zeiss). The starting time point (0 min) was set as 15 min before cleavage furrow formation when cells were still round. The cells were tracked until the incision between daughter cells was observed. Cytokinesis duration was calculated from the starting time point to the incision time point in each cell. Serial images were compiled in JPEG format and exported to Adobe Photoshop for processing.

### Purification of recombinant GST- and His-tagged fusion proteins

BL21 (DE3) *Escherichia coli* (Invitrogen) containing pDEST15 or pDEST17 plasmids encoding the indicated CENP-E fragments and full-length PRC1 were cultured in LB medium until OD_600_ = 0.5. Isopropyl-β-D-thiogalactopyranoside was then added to a final concentration of 0.2 mM and cells were incubated overnight at 25°C for protein expression. Affinity purification of GST- and His-tagged fusion proteins was performed using the B-PER GST Fusion Protein Purification Kit (Thermo Scientific) and QIAexpress Ni-NTA Fast Start Kit (Qiagen), respectively, according to the manufacturers' instructions.

### CENP-E enzyme assay

The CENP-E ATPase assay was performed using the Kinesin ATPase End Point Biochem Kit (Cytoskeleton, Inc., Denver, CO, USA) according to the manufacturer's instructions. The CENP-E motor domain was purchased from Cytoskeleton, Inc. The ATPase assay for human CENP-E activity was performed using 6.7 μg/mL of the CENP-E motor domain, 66.7 μg/mL microtubules (Cytoskeleton Inc.), 300 μM ATP, and the indicated GST-fusion proteins (0, 0.5, 1.25, and 2.5 μg). Reactions were performed in 30 μL of reaction buffer for 5 min at room temperature. The ATPase reaction was determined by a spectrophotometer at OD = 650 nm.

### Peptide synthesis

A peptide conjugated with a cell membrane-permeable polyarginine residue (Arg^11^) at the N-terminus (RRRRRRRRRRR-GGG-VRYFDNSSLG) and a control peptide (RRRRRRRRRRR-GGG) were synthesized by Scrum, Inc. (Tokyo, Japan).

## SUPPLEMENTARY FIGURES AND TABLE



## References

[1] Morgan DO (2007). The Cell Cycle.

[2] Steigemann P, Gerlich DW (2009). Cytokinetic abscission: cellular dynamics at the midbody. Trends Cell Biol.

[3] Glotzer M (2005). The molecular requirements for cytokinesis. Science.

[4] Glotzer M (2009). The 3Ms of central spindle assembly: microtubules, motors and MAPs. Nat Rev Mol Cell Biol.

[5] Storchova Z, Pellman D (2004). From polyploidy to aneuploidy, genome instability and cancer. Nat Rev Mol Cell Biol.

[6] Skop AR, Liu H, Yates J, Meyer BJ, Heald R (2004). Dissection of the mammalian midbody proteome reveals conserved cytokinesis mechanisms. Science.

[7] Subramanian R, Wilson-Kubalek EM, Arthur CP, Bick MJ, Campbell EA, Darst SA, Milligan RA, Kapoor TM (2010). Insights into antiparallel microtubule crosslinking by PRC1, a conserved nonmotor microtubule binding protein. Cell.

[8] Walczak CE, Shaw SL (2010). A MAP for bundling microtubules. Cell.

[9] Kurasawa Y, Earnshaw WC, Mochizuki Y, Dohmae N, Todokoro K (2004). Essential roles of KIF4 and its binding partner PRC1 in organized central spindle midzone formation. EMBO J.

[10] Mollinari C, Kleman JP, Saoudi Y, Jablonski SA, Perard J, Yen TJ, Margolis RL (2005). Ablation of PRC1 by small interfering RNA demonstrates that cytokinetic abscission requires a central spindle bundle in mammalian cells, whereas completion of furrowing does not. Molecular biology of the cell.

[11] Bieling P, Telley IA, Surrey T (2010). A minimal midzone protein module controls formation and length of antiparallel microtubule overlaps. Cell.

[12] Hu CK, Coughlin M, Field CM, Mitchison TJ (2011). KIF4 regulates midzone length during cytokinesis. Curr Biol.

[13] Nunes Bastos R, Gandhi SR, Baron RD, Gruneberg U, Nigg EA, Barr FA (2013). Aurora B suppresses microtubule dynamics and limits central spindle size by locally activating KIF4A. The Journal of cell biology.

[14] Bastos RN, Cundell MJ, Barr FA (2014). KIF4A and PP2A-B56 form a spatially restricted feedback loop opposing Aurora B at the anaphase central spindle. The Journal of cell biology.

[15] Miki H, Okada Y, Hirokawa N (2005). Analysis of the kinesin superfamily: insights into structure and function. Trends Cell Biol.

[16] Yen TJ, Li G, Schaar BT, Szilak I, Cleveland DW (1992). CENP-E is a putative kinetochore motor that accumulates just before mitosis. Nature.

[17] Wood KW, Sakowicz R, Goldstein LS, Cleveland DW (1997). CENP-E is a plus end-directed kinetochore motor required for metaphase chromosome alignment. Cell.

[18] Yao X, Anderson KL, Cleveland DW (1997). The microtubule-dependent motor centromere-associated protein E (CENP-E) is an integral component of kinetochore corona fibers that link centromeres to spindle microtubules. The Journal of cell biology.

[19] Kapoor TM, Lampson MA, Hergert P, Cameron L, Cimini D, Salmon ED, McEwen BF, Khodjakov A (2006). Chromosomes can congress to the metaphase plate before biorientation. Science.

[20] Cai S, O'Connell CB, Khodjakov A, Walczak CE (2009). Chromosome congression in the absence of kinetochore fibres. Nature cell biology.

[21] Barisic M, Silva e Sousa R, Tripathy SK, Magiera MM, Zaytsev AV, Pereira AL, Janke C, Grishchuk EL, Maiato H (2015). Mitosis. Microtubule detyrosination guides chromosomes during mitosis. Science.

[22] Yen TJ, Compton DA, Wise D, Zinkowski RP, Brinkley BR, Earnshaw WC, Cleveland DW (1991). CENP-E, a novel human centromere-associated protein required for progression from metaphase to anaphase. EMBO J.

[23] Liu D, Zhang N, Du J, Cai X, Zhu M, Jin C, Dou Z, Feng C, Yang Y, Liu L, Takeyasu K, Xie W, Yao X (2006). Interaction of Skp1 with CENP-E at the midbody is essential for cytokinesis. Biochem Biophys Res Commun.

[24] Wood KW, Lad L, Luo L, Qian X, Knight SD, Nevins N, Brejc K, Sutton D, Gilmartin AG, Chua PR, Desai R, Schauer SP, McNulty DE, Annan RS, Belmont LD, Garcia C (2010). Antitumor activity of an allosteric inhibitor of centromere-associated protein-E. Proceedings of the National Academy of Sciences of the United States of America.

[25] Schaar BT, Chan GK, Maddox P, Salmon ED, Yen TJ (1997). CENP-E function at kinetochores is essential for chromosome alignment. The Journal of cell biology.

[26] Yao X, Abrieu A, Zheng Y, Sullivan KF, Cleveland DW (2000). CENP-E forms a link between attachment of spindle microtubules to kinetochores and the mitotic checkpoint. Nature cell biology.

[27] Putkey FR, Cramer T, Morphew MK, Silk AD, Johnson RS, McIntosh JR, Cleveland DW (2002). Unstable kinetochore-microtubule capture and chromosomal instability following deletion of CENP-E. Developmental cell.

[28] Mao Y, Desai A, Cleveland DW (2005). Microtubule capture by CENP-E silences BubR1-dependent mitotic checkpoint signaling. The Journal of cell biology.

[29] Weaver BA, Cleveland DW (2007). Aneuploidy: instigator and inhibitor of tumorigenesis. Cancer research.

[30] Morishita D, Takami M, Yoshikawa S, Katayama R, Sato S, Kukimoto-Niino M, Umehara T, Shirouzu M, Sekimizu K, Yokoyama S, Fujita N (2011). Cell-permeable carboxyl-terminal p27(Kip1) peptide exhibits anti-tumor activity by inhibiting Pim-1 kinase. The Journal of biological chemistry.

[31] Hu CK, Coughlin M, Mitchison TJ (2012). Midbody assembly and its regulation during cytokinesis. Molecular biology of the cell.

[32] Ohashi A, Ohori M, Iwai K, Nakayama Y, Nambu T, Morishita D, Kawamoto T, Miyamoto M, Hirayama T, Okaniwa M, Banno H, Ishikawa T, Kandori H, Iwata K (2015). Aneuploidy generates proteotoxic stress and DNA damage concurrently with p53-mediated post-mitotic apoptosis in SAC-impaired cells. Nature communications.

[33] Ohashi A, Ohori M, Iwai K, Nambu T, Miyamoto M, Kawamoto T, Okaniwa M (2015). A Novel Time-Dependent CENP-E Inhibitor with Potent Antitumor Activity. PLoS One.

[34] Garcia-Saez I, Yen T, Wade RH, Kozielski F (2004). Crystal structure of the motor domain of the human kinetochore protein CENP-E. J Mol Biol.

[35] Elia N, Ott C, Lippincott-Schwartz J (2013). Incisive imaging and computation for cellular mysteries: lessons from abscission. Cell.

[36] Mollinari C, Kleman JP, Jiang W, Schoehn G, Hunter T, Margolis RL (2002). PRC1 is a microtubule binding and bundling protein essential to maintain the mitotic spindle midzone. The Journal of cell biology.

[37] Liao H, Li G, Yen TJ (1994). Mitotic regulation of microtubule cross-linking activity of CENP-E kinetochore protein. Science.

[38] Gruneberg U, Neef R, Li X, Chan EH, Chalamalasetty RB, Nigg EA, Barr FA (2006). KIF14 and citron kinase act together to promote efficient cytokinesis. The Journal of cell biology.

[39] Lee KY, Esmaeili B, Zealley B, Mishima M (2015). Direct interaction between centralspindlin and PRC1 reinforces mechanical resilience of the central spindle. Nature communications.

[40] Zhu C, Jiang W (2005). Cell cycle-dependent translocation of PRC1 on the spindle by Kif4 is essential for midzone formation and cytokinesis. Proceedings of the National Academy of Sciences of the United States of America.

[41] Rusten TE, Vaccari T, Stenmark H (2012). Shaping development with ESCRTs. Nature cell biology.

[42] Schiel JA, Prekeris R (2013). Membrane dynamics during cytokinesis. Current opinion in cell biology.

[43] Steigemann P, Wurzenberger C, Schmitz MH, Held M, Guizetti J, Maar S, Gerlich DW (2009). Aurora B-mediated abscission checkpoint protects against tetraploidization. Cell.

[44] Ohashi A, Zdzienicka MZ, Chen J, Couch FJ (2005). Fanconi anemia complementation group D2 (FANCD2) functions independently of BRCA2- and RAD51-associated homologous recombination in response to DNA damage. The Journal of biological chemistry.

