# Supplementary material for: Motor activity of centromere-associated protein-E contributes to its localization at the center of the midbody to regulate cytokinetic abscission
Source: Oncotarget. 2016 Nov 8;7(48):79964–80. doi: 10.18632/oncotarget.13206 (PMC5346764; doi:10.18632/oncotarget.13206)
Supplement: Supplementary file 1 [file oncotarget-07-79964-s001.pdf]

Motor activity of centromere-associated protein-E contributes to its localization at the center of the midbody to regulate cytokinetic abscission

SUPPLEMENTARY FIGURES AND TABLE

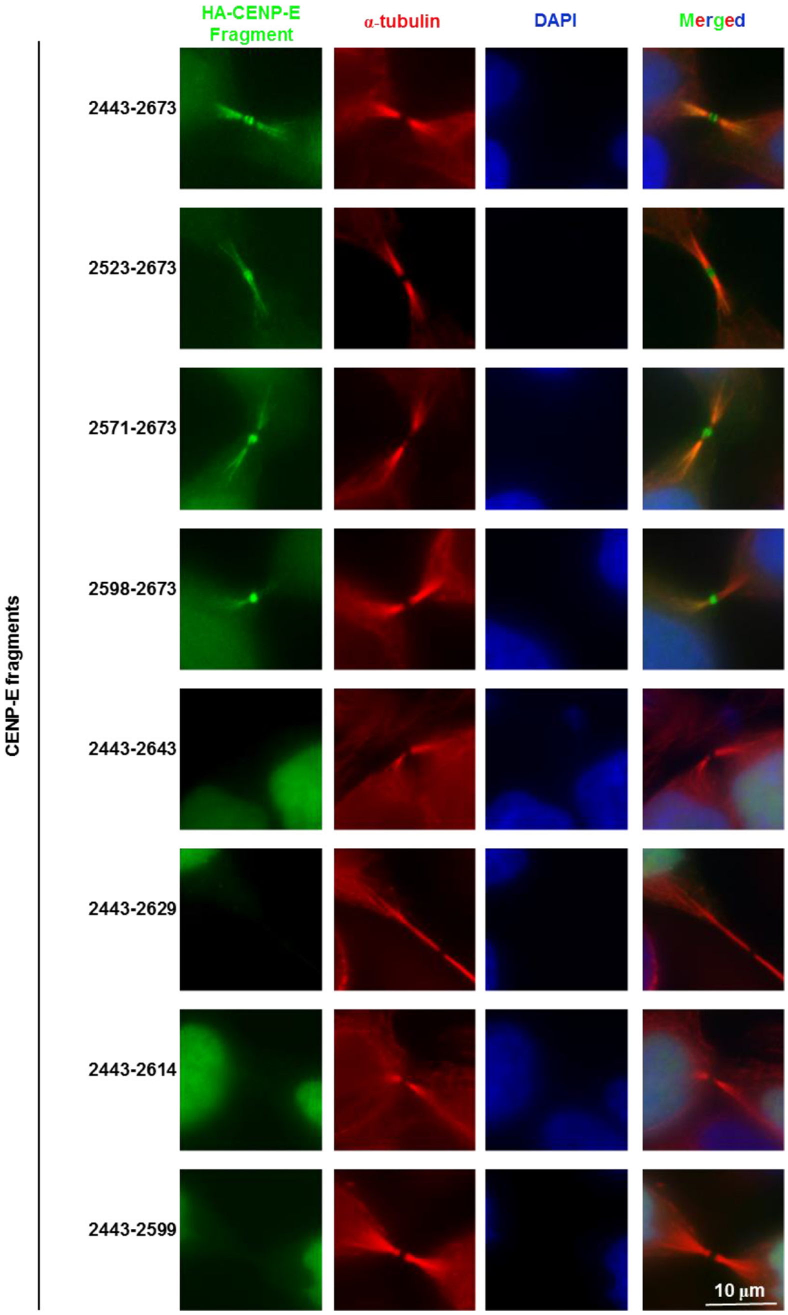

Supplementary Figure S1: Representative images of HA-tagged CENP-E fragments localized to the midbody. The indicated HA-tagged CENP-E fragments were transfected into HeLa cells. The cells were fixed for immunofluorescence 24 h after plasmid transfection. Representative images show cellular localization of CENP-E fragments at telophase. Green and red signals indicate HA-tagged CENP-E fragments and anti-α-tubulin, respectively. DNA was labeled with DAPI (blue). White bar indicates 10 μm.

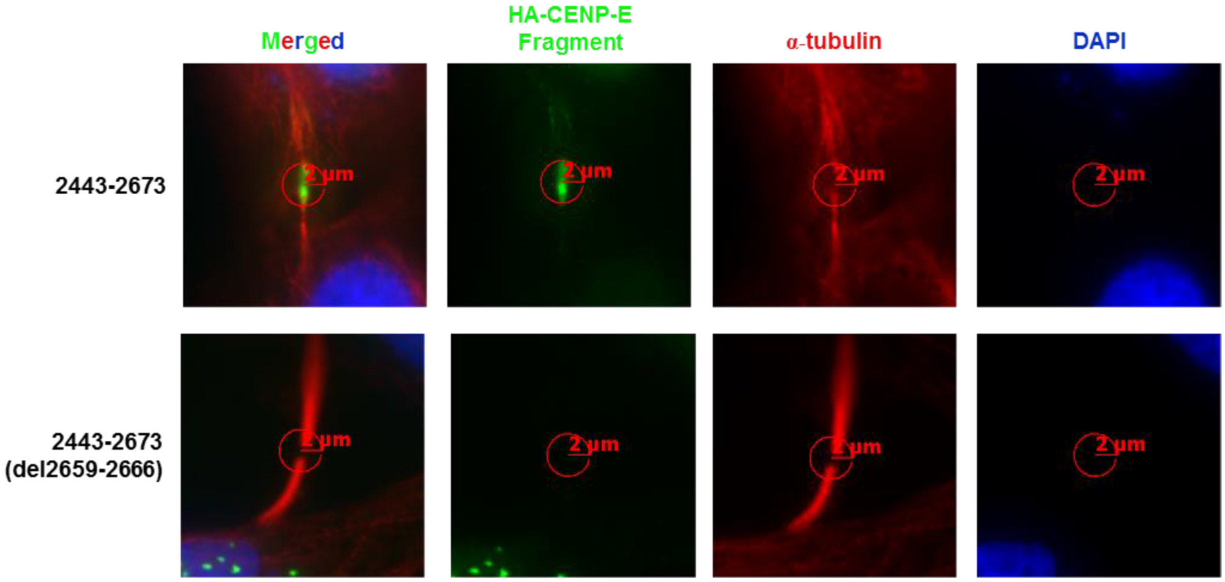

Supplementary Figure S2: Quantification of HA-tagged CENP-E fragments at the midbody. Representative images showing localization of HA-tagged CENP-E fragments at the midbody. The indicated HA-tagged CENP-E fragments were transfected into HeLa cells. The cells were fixed for immunofluorescence 24 h after plasmid transfection. Green and red signals indicate HA-tagged CENP-E fragments and anti-α-tubulin, respectively. For each fragment, the green fluorescence intensities within a circle of 2-µm radius at the midbody were normalized to the mean intensity of cells transfected with fragment 2443–2673 (Δ2659–2666).

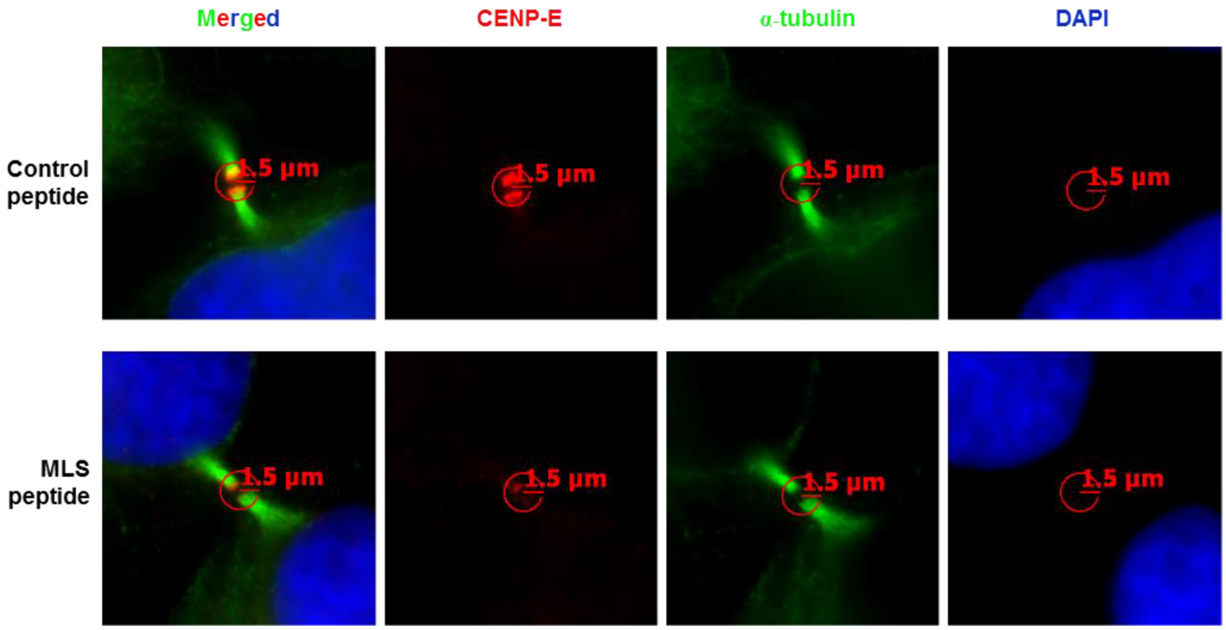

Supplementary Figure S3: Quantification of endogenous CENP-E at the midbody of MLS-treated cells. Representative images of endogenous CENP-E at the midbody. HeLa cells were treated with the MLS or control peptide for 24 h. Red and green signals indicate CENP-E and anti-α-tubulin, respectively. The red fluorescence intensity within a circle of 1.5-µm radius at the midbody of MLS-treated cells was normalized to the mean intensity of control peptide-treated cells.

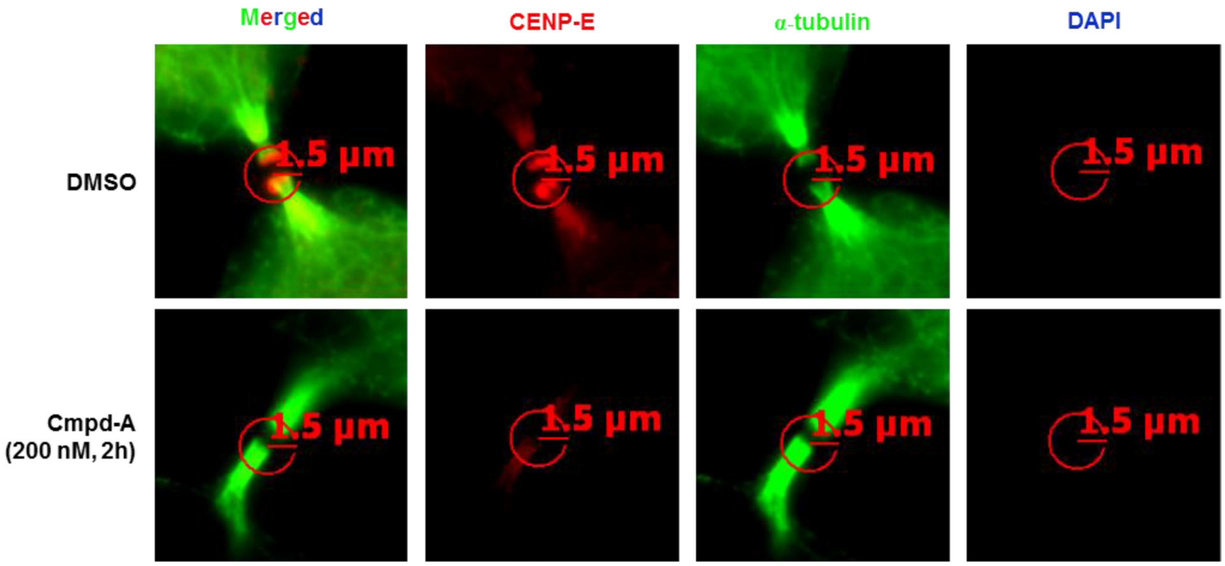

Supplementary Figure S4: Quantification of endogenous CENP-E in Cmpd-A-treated cells. Representative images of endogenous CENP-E at midbody. HeLa cells were treated with Cmpd-A or DMSO for 2 h. Red and green signals indicate CENP-E and anti-α-tubulin, respectively. The red fluorescent intensity in a circle of 1.5-μm radius was normalized to the mean intensity of DMSO-treated cells.

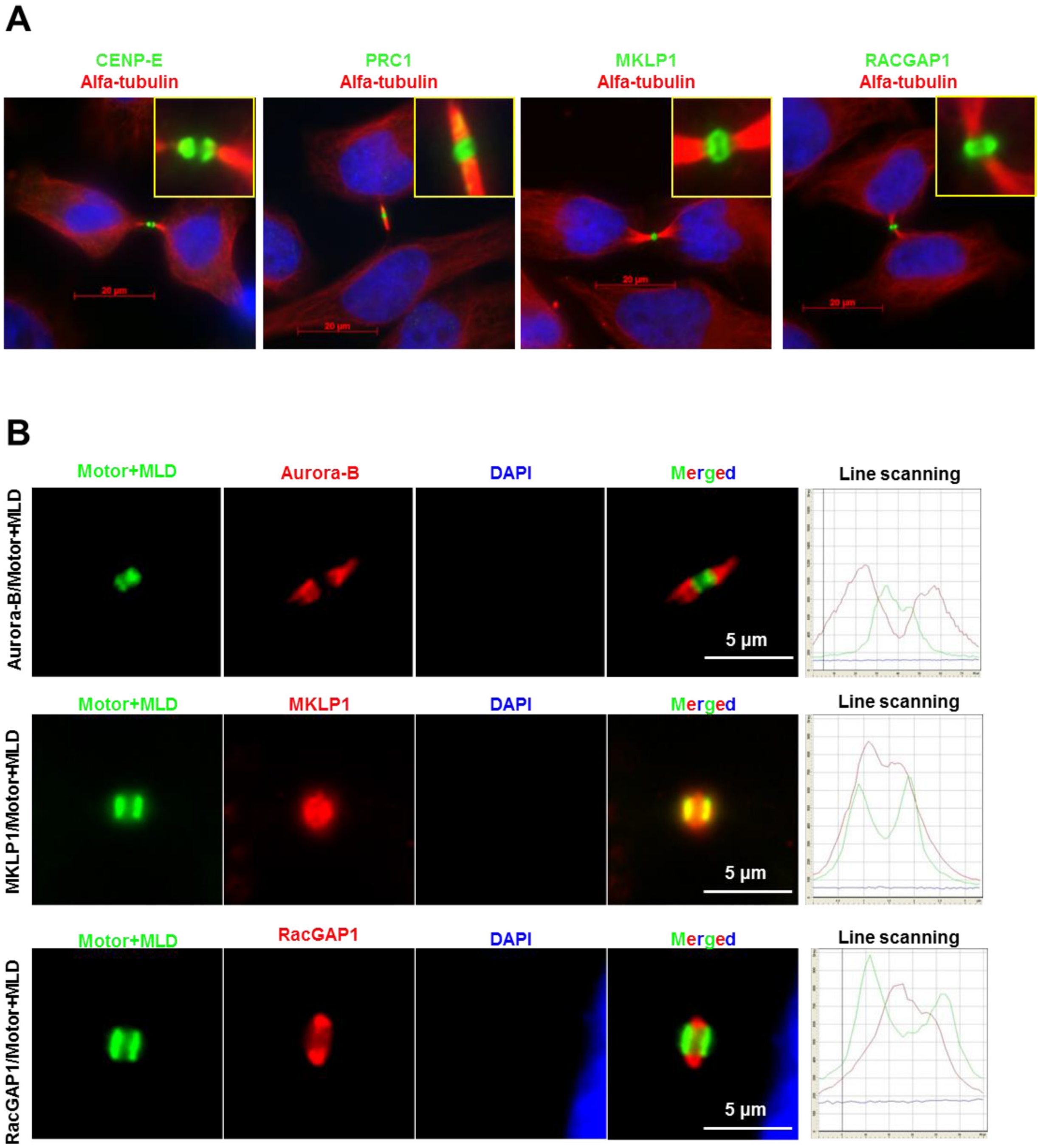

Supplementary Figure S5: Motor+MLD and PRC1 are colocalized at the midbody in HeLa cells. A. Endogenous CENP-E localizes at the bulge of the midbody. Cellular localization of CENP-E in HeLa cells at telophase by immunofluorescence with anti-CENP-E, PRC1, MKLP1, and RacGAP1 (green). Red and blue signals indicate α-tubulin and DAPI, respectively. **B.** Co-immunofluorescence of Motor+HA-MLD and endogenous aurora-A, MKLP1, and RacGAP1. Green signals indicate HA-Motor+MLD. Red signals indicate aurora-A, (upper) MKLP1 (middle), and RacGAP1 (lower). White bars indicate 5 μm. Histogram of the line scanning indicates the intensity of each signal.

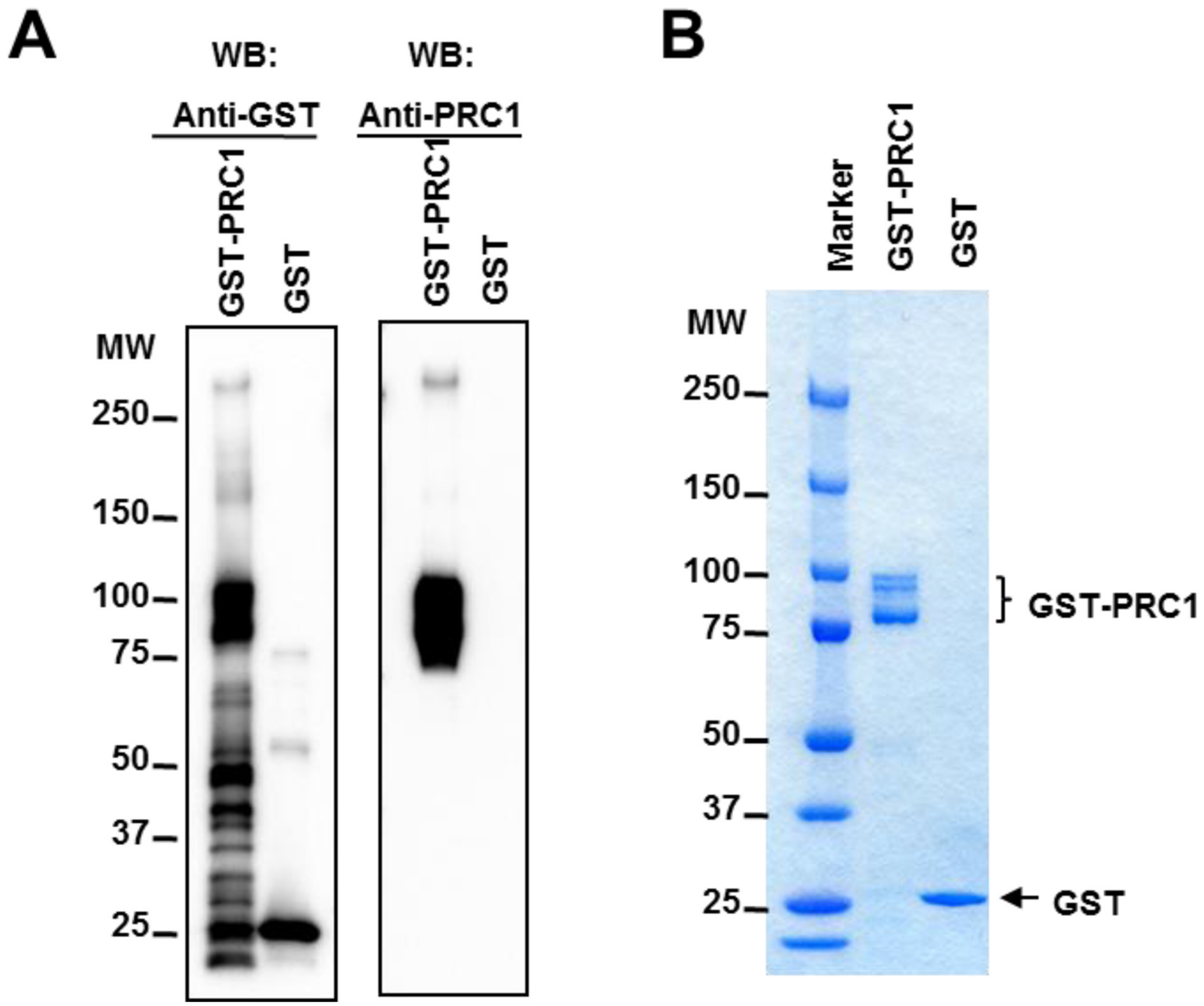

Supplementary Figure S6: Recombinant GST-fusion PRC1 protein. A. GST-PRC1 and GST proteins immobilized on glutathione-sepharose beads were analyzed by immunoblotting with anti-GST and anti-PRC1 antibodies. **B.** GST-PRC1 and GST proteins immobilized on glutathione-sepharose beads were analyzed by Coomassie Brilliant Blue (CBB) staining.

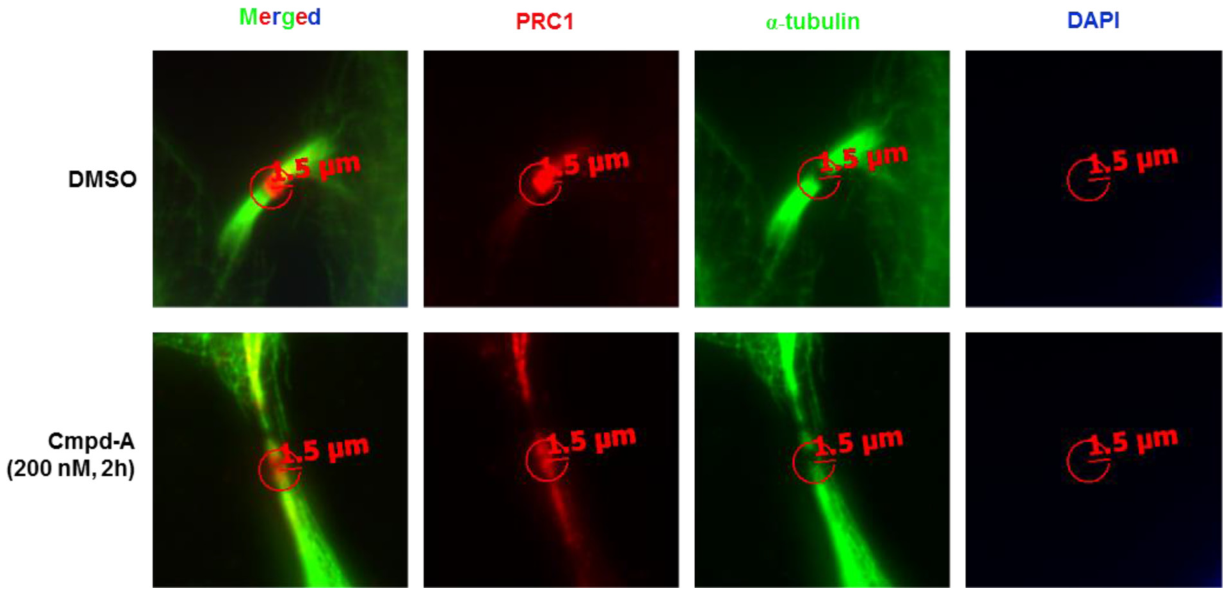

Supplementary Figure S7: Quantification of endogenous PRC1 in Cmpd-A-treated cells. Representative images of endogenous PRC1 at the midbody. HeLa cells were treated with Cmpd-A or DMSO for 2 h. Red and green signals indicate PRC1 and anti-α-tubulin, respectively. The red fluorescence intensity within a circle of 1.5-μm radius was normalized to the mean intensity of DMSO-treated cells.

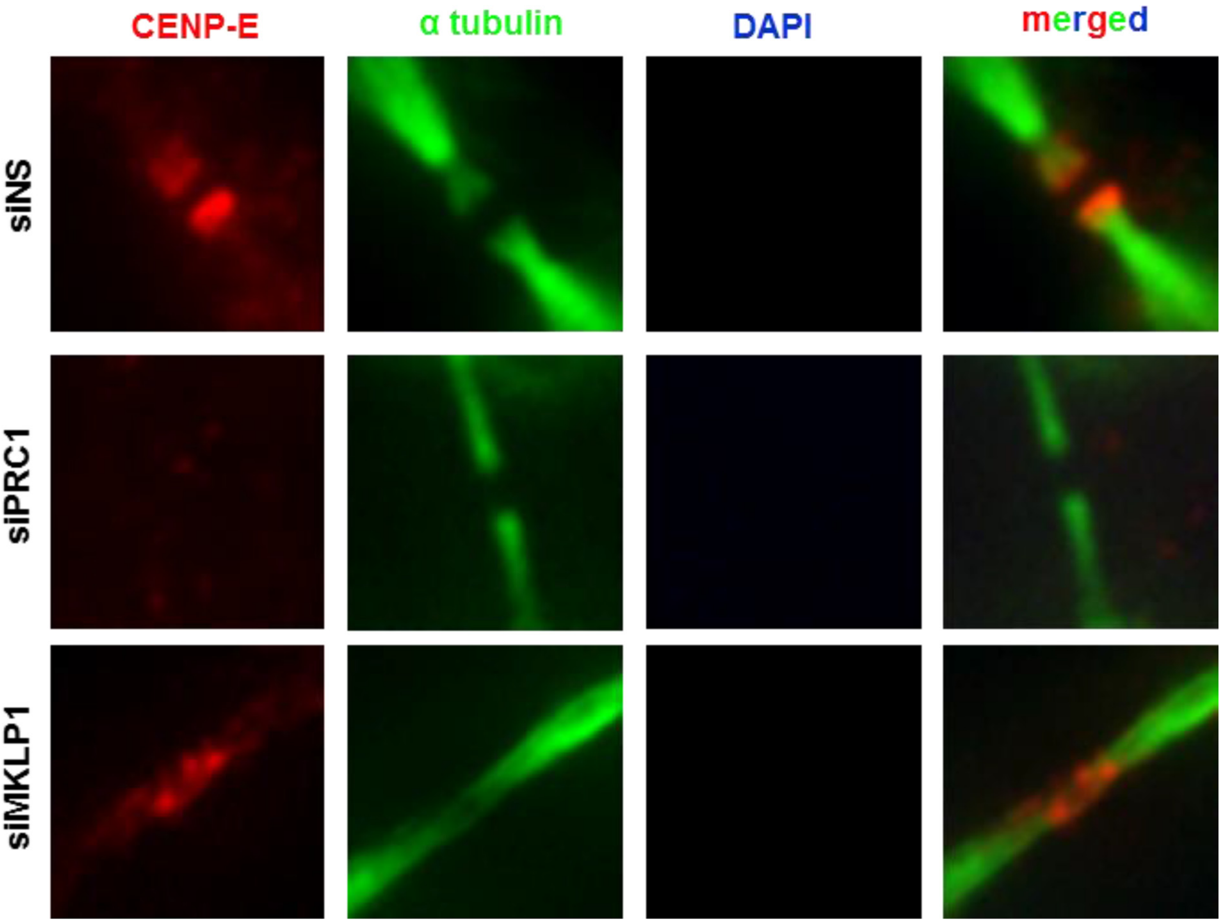

Supplementary Figure S8: PRC1 knockdown attenuated localization of endogenous CENP-E at the midbody. HeLa cells were treated with siNS (upper), siPRC1 (middle), or siMKLP1 (lower). Twenty-four hours after transfection, the cells were fixed for immunofluorescence. Immunofluorescence was performed with anti-CENP-E (red) and anti-a-tubulin (green). DNA was labeled with DAPI (blue).

Supplementary Table S1: Primers used in this study

Primer ID	Primer Sequence (Bold: target sequence, Fine: Gateway Sequence)
CENP-E_1F_Gateway	5'-GGGGACAAGTTTGTACAAAAAAGCAGGCTGC**ATGGCGGAGGAAGGAGCCGT-3'**
CENP-E_400R_Gateway	5'-GGGGACCACTTTGTACAAGAAAGCTGGGTC**CATCCGTGTTAAGTTTTCAA-3'**
CENP-E_397F_Gateway	5'-GGGGACAAGTTTGTACAAAAAAGCAGGCTGC**TTAACACGGATGCTGGTGAC-3'**
CENP-E_993R_Gateway	5'-GGGGACCACTTTGTACAAGAAAGCTGGGTC**CCTGGAAACTTCCTCAGAAA-3'**
CENP-E_988F_Gateway	5'-GGGGACAAGTTTGTACAAAAAAGCAGGCTGC**TCTGAGGAAGTTTCCAGGAA-3'**
CENP-E_1584R_Gateway	5'-GGGGACCACTTTGTACAAGAAAGCTGGGTC**TTCTTCTTGACTTTCTTGAA-3'**
CENP-E_1579F_Gateway	5'-GGGGACAAGTTTGTACAAAAAAGCAGGCTGC**CAAGAAAGTCAAGAAGAAAT-3'**
CENP-E_2137R_Gateway	5'-GGGGACCACTTTGTACAAGAAAGCTGGGTC**TCTCATTGAAAGCTCTTTTT-3'**
CENP-E_2357F_Gateway	5'-GGGGACAAGTTTGTACAAAAAAGCAGGCTGC**GGTGCCCAGGTTAATCCTACCA-3'**
CENP-E_2701R_Gateway	5'-GGGGACCACTTTGTACAAGAAAGCTGGGTC**CTACTGAGTTTTGCACTCAGGCA-3'**
CENP-E_2443F_Gateway	5'-GGGGACAAGTTTGTACAAAAAAGCAGGCTGC**CAGGACAAAGTTGCTTTAGG-3'**
CENP-E_2673R_Gateway	5'-GGGGACCACTTTGTACAAGAAAGCTGGGTC**TTGCACCTCTGGACAAAGGC-3'**
CENP-E_2523F_Gateway	5'-GGGGACAAGTTTGTACAAAAAAGCAGGCTGC**CCTTCAAATAAACCCTTAAC-3'**
CENP-E_2571F_Gateway	5'-GGGGACAAGTTTGTACAAAAAAGCAGGCTGC**AATGAATTGTTAAGCAATAA-3'**
CENP-E_2598F_Gateway	5'-GGGGACAAGTTTGTACAAAAAAGCAGGCTGC**AAACAAGTAACTTGTGAGAA-3'**
CENP-E_2643R_Gateway	5'-GGGGACCACTTTGTACAAGAAAGCTGGGTC**ACAAGATTTTGGTGATTCCT-3'**
CENP-E_2629R_Gateway	5'-GGGGACCACTTTGTACAAGAAAGCTGGGTC**CCGTTCCTTGCATTGAGAGG-3'**
CENP-E_2614R_Gateway	5'-GGGGACCACTTTGTACAAGAAAGCTGGGTC**TGTTCCAGTCACTTTAGGAG-3'**
CENP-E_2600R_Gateway	5'-GGGGACCACTTTGTACAAGAAAGCTGGGTC**TACTTGTTTGTGAGCCTCTC-3'**
CENP-E_del.2641-2651F	**5'-AATCACCATTACCATCACCTCATCCA-3'**
CENP-E_del.2641-2651R	**5'-ATGGTAATGGTGATTCCTTTGGCAC-3'**
CENP-E_del.2648-2658F	**5'-TGATAGCCGCTATTTTGATAACTCA-3'**
CENP-E_del.2648-2658R	**5'-AAATAGCGGCTATCAAAAAAACAAGA-3'**
CENP-E_del.2659-2666F	**5'-ATCCAGTTGGCCTTTGTCCAGAGGTG-3'**
CENP-E_del.2659-2666R	**5'-AAAGGCCAACTGGATGAGGTGATGG-3'**
